# Genetic Inactivation of *Chlamydia trachomatis* Inclusion Membrane Protein CT228 Alters MYPT1 Recruitment, Extrusion Production, and Longevity of Infection

**DOI:** 10.3389/fcimb.2018.00415

**Published:** 2018-11-30

**Authors:** Jennifer H. Shaw, Charlotte E. Key, Timothy A. Snider, Prakash Sah, Edward I. Shaw, Derek J. Fisher, Erika I. Lutter

**Affiliations:** ^1^Department of Integrative Biology, Oklahoma State University, Stillwater, OK, United States; ^2^Department of Veterinary Pathobiology, Oklahoma State University, Stillwater, OK, United States; ^3^Department of Microbiology and Molecular Genetics, Oklahoma State University, Stillwater, OK, United States; ^4^Department of Microbiology, Southern Illinois University, Carbondale, IL, United States

**Keywords:** *Chlamydia*, extrusion, lymphogranuloma venereum, L2 serovar, sexually transmitted infection, urogenital infection, CT228, mouse infection

## Abstract

*Chlamydia trachomatis* is an obligate intracellular pathogen with global health and economic impact. Upon infection, *C. trachomatis* resides within a protective niche, the inclusion, wherein it replicates and usurps host cell machinery and resources. The inclusion membrane is the key host-pathogen interface that governs specific protein-protein interactions to manipulate host signaling pathways. At the conclusion of the infection cycle, *C. trachomatis* exits the host cell via lysis or extrusion. Extrusion depends on the phosphorylation state of myosin light chain 2 (MLC2); the extent of phosphorylation is determined by the ongoing opposing activities of myosin phosphatase (MYPT1) and myosin kinase (MLCK). Previously, it was shown that MYPT1 is recruited to the inclusion and interacts with CT228 for regulation of host cell egress. In this study, we generated a targeted chromosomal mutation of *CT228* (L2-ΔCT228) using the TargeTron system and demonstrate a loss of MYPT1 recruitment and increase in extrusion production *in vitro*. Mutation of *CT228* did not affect chlamydial growth in cell culture or recruitment of MLC2. Moreover, we document a delay in clearance of L2-ΔCT228 during murine intravaginal infection as well as a reduction in systemic humoral response, relative to L2-wild type. Taken together, the data suggest that loss of MYPT1 recruitment (as a result of CT228 disruption) regulates the degree of host cell exit via extrusion and affects the longevity of infection *in vivo*.

## Introduction

*Chlamydia trachomatis* (serovars D-K, L1-L3) is the most commonly reported bacterial sexually transmitted infection (STI) in the United States and worldwide with over 130 million new cases each year (Newman et al., 2015). While antibiotic therapy is efficacious, re-infection is common and many chlamydial infections are asymptomatic thereby left untreated. Ongoing infection in women leads to scarring within the reproductive tract, which may cause pelvic inflammatory disease, ectopic pregnancy and infertility. Likewise, extensive scarring in response to ocular infection (serovars A–C) is the leading cause of preventable blindness in underdeveloped countries (Bourne et al., 2013). The global impact on health and economic burden necessitates the development of a vaccine. Vaccine strategies were initially investigated in the 1960s wherein short term immunity was produced against ocular trachoma in children vaccinated using live or fixed whole bacteria (Grayston et al., 1963; Dhir et al., 1967) and more recently, a live-attenuated vaccine protected non-human primates against trachoma (Kari et al., 2011). Currently, there are five vaccine candidates in preclinical trials and one (MOMP-VD4) is in Phase I (Poston et al., 2017). While significant progress has been achieved, a major limitation to elucidating the drivers of pathogenesis and long-term immunity has been the prior lack of genetic tools.

The technical challenge in studying chlamydial pathogenesis is largely due to the complex nature of this obligate intracellular pathogen which exhibits a biphasic development cycle involving an infectious form, the elementary body (EB), and a non-infectious replicative form termed a reticulate body (RB) (Moulder, 1991). Upon infection, *Chlamydia* directs the formation of a protective niche within the host cell called an inclusion, which becomes decorated with *Chlamydia-*derived inclusion membrane proteins (Incs) that interface with the host cytosol (Hackstadt et al., 1997) as well as kinase laden microdomain regions where inclusion membrane proteins interact with the host cytoskeleton and signaling pathways (Mital et al., 2010; Mital and Hackstadt, 2011). Within this specialized vacuole, EBs differentiate into RBs followed by abundant replication of RBs causing the inclusion to enlarge. Within 24–48 h the RBs undergo asynchronous differentiation back to EBs in preparation for dissemination while avoiding detection by the host innate immune response. Host cell egress occurs either by lysis of the infected cell releasing EBs or extrusion of EB-filled inclusions wrapped in host membrane leaving behind an intact host cell (Doughri et al., 1972; Hybiske and Stephens, 2007, 2008).

The extrusion exit mechanism may provide a means to temporarily evade the host immune response via enclosure within host membrane and maximize dissemination by delivery of *Chlamydia*-rich vesicles to neighboring cells rather than individual EBs. Uptake of extrusions by macrophages and survival within has been documented *in vitro* (Zuck et al., 2016a), however whether this occurs *in vivo* remains unknown. While there may be benefits to the pathogen for this exit strategy, unbridled extrusion would likely be accompanied by a substantial metabolic cost to its host. Studies show that extrusion is negatively regulated by the interaction between *C. trachomatis* inclusion membrane protein CT228 and host myosin phosphatase target subunit 1 (MYPT1). Whether *Chlamydia* exit via lysis or extrusion is controlled by the phosphorylation state of myosin light chain 2 (MLC2): dephosphorylated MLC2 favors lysis while phosphorylated MLC2 enhances extrusion (Lutter et al., 2013). Therefore, recruitment of host MYPT1 to the inclusion by CT228 serves to regulate the manner and degree of host cell egress. Although the extrusion process has been observed *in vitro* for many years (Todd and Caldwell, 1985; Hybiske and Stephens, 2007, 2008), it was only recently documented to occur *in vivo* across multiple serovars (Shaw et al., 2017).

In the last 8 years genetic approaches have proven feasible across chlamydial species and serovars enabling the generation of targeted chlamydial mutants and expression of recombinant genes (Hooppaw and Fisher, 2015). These advances have been crucial in confirming the secretion and localization of *C. trachomatis* Incs (Weber et al., 2015) and open the path to discovery of virulence factors. Herein, we utilized the TargeTron^TM^ method (Johnson and Fisher, 2013) to produce a targeted chromosomal mutation in *C. trachomatis* serovar L2 inclusion membrane protein CT228 (L2-ΔCT228). We report that the resultant loss of CT228 did not affect chlamydial growth in cell culture. However, recruitment of MYPT1 to the inclusion membrane failed to occur and a significant increase in extrusions was observed *in vitro*. Moreover, we documented a delay in clearance of L2-ΔCT228 during murine intravaginal infection, suggesting that enhanced host cell exit of EBs within the protective enclosure of an extrusion may reduce antigen presentation to the host immune system. This is further supported by a significant reduction in mean systemic anti-*Chlamydia* IgG2a titers following infection with L2-ΔCT228 compared to the L2-wild type.

## Methods

### Chlamydial strains and cell culture

*Chlamydia trachomatis* serovar L2-wild type and L2-ΔCT228 (LGV 434/Bu, originating from the Hackstadt Lab at Rocky Mountain Laboratories, Hamilton, MT) were propagated in HeLa 229 cells and purified by Renografin density gradient centrifugation as previously described (Caldwell et al., 1981). HeLa were grown in RPMI 1640 + 5% fetal bovine serum (FBS) at 37°C with 5% CO_2_ and 1 μg/mL of cycloheximide added as needed.

### Construction of pDFTT295 and selection of a *C. trachomatis* L2-ΔCT228

The chlamydial TargeTron vector pDFTT3(bla) was modified to allow for spectinomycin selection by replacing the *bla* cassette with the *aadA* cassette (Lowden et al., 2015). The TargeTron algorithm from Sigma-Aldrich was used to predict CT228 insertion sites and primers were chosen that would target intron insertion at bp 295 (A in ATG used as position 1) in an antisense orientation (E-value of 0.200). The vector was then retargeted for insertion into *CT228* using PCR with Phusion High-Fidelity PCR Master Mix (Thermo-Fisher) and primers 228A, 228B, 228C, and EBS universal (Integrated DNA Technologies, Coralville, IA; Table S1) followed by DNA restriction digestion as previously described (Johnson and Fisher, 2013). The pDFTT295 vector was propagated in *Escherichia coli* DH5α grown at 30°C in LB supplemented with 20 μg/ml chloramphenicol. *Chlamydia trachomatis* transformation and mutant selection was carried out as described in Lowden et al. using 500 μg/ml spectinomycin (Lowden et al., 2015). The passage 4 mutants were plaque purified to obtain a clonal L2-ΔCT228 strain. The plaque isolate was serially expanded in cell culture using HeLa cells and stored in sucrose-phosphate-glutamic acid (SPG) buffer at −80°C. Stocks were titered using the infectious progeny quantitation assay.

### PCR validation of L2-ΔCT228

Genomic DNA was harvested from L2-wild type and L2-ΔCT228 EB stocks using the DNeasy Blood and Tissue Kit from Qiagen. PCR reactions were performed with 50 ng of genomic DNA or 5 ng of plasmid DNA and Phusion High-Fidelity PCR Master Mix. The primers used are provided in Table S1. PCR products were run on 1% agarose gels, stained with ethidium bromide, and viewed using UV transillumination. To confirm the location of the GII insertion in *CT228*, the PCR product generated from primers CT228-2F and CT228-2R was extracted from the agarose gel using the GeneJET gel extraction kit (Thermo-Fisher) and ligated into pJET (Thermo-Fisher) to generate pJET::CT228GII*aadA*. Ligation products were transformed into *E. coli* DH5α and transformants were selected at 37°C on Luria-Bertani (LB) agar plates supplemented with 100 μg/ml of ampicillin. A colony was then selected for overnight growth in LB with ampicillin and the plasmid was extracted using the GeneJET Plasmid Miniprep kit (Thermo-Fisher). The gene insert in pJET::CT228GIIaadA was sequenced using the vector specific primers JETF and JETR via Sanger sequencing performed by Macrogen USA. Sequencing results were analyzed using BioEdit (TA Hall 1999) and Clone Manager (Sci-Ed Software).

### Transcript analysis of the L2-wild type and L2-ΔCT228 *CT229-CT224* gene linkage group

Total RNA was harvested from *C. trachomatis* serovar L2-wild type and L2-ΔCT228 (LGV 434) infected Hela cells at 24 h post-infection using Tri Reagent (Ambion, Austin, TX). To remove contaminating DNA, all RNA samples were treated with RQ1 DNase (Promega, Madison, WI). Removal of contaminating DNA was confirmed using PCR. Reverse Transcriptase (RT)-PCR analysis was carried out using the Access Quick RT-PCR Kit (Promega, Madison, WI) following the manufacturer's instructions. All oligonucleotide primers used in this study are shown in Table S1. RT-PCR analysis of *groEL* (control), *CT229, CT228, CT227, CT226, CT225*, and *CT224* open reading frames (ORFs) was performed using oligonucleotide primer pairs within each ORF, respectively. For CT228, primers pairs designed upstream (5′), downstream (3′), or spanning the TargeTron insert were employed. Amplicons were separated by 1.5% agarose gel electrophoresis. Ethidium bromide stained gels were visualized on a BioRad Imager (BioRad, Hercules, CA). Genomic DNA samples were analyzed in parallel during each assay as a PCR positive control.

### Growth and infectious progeny quantitation

*Chlamydia trachomatis* L2-wild type and L2-ΔCT228 were used to infect HeLa cell monolayers at 90% confluency in 24 well plates at a MOI of ~0.5; inoculated plates were centrifuged for 1 h at 700 × g then any free EBs were washed away prior to feeding cells. At 0, 6, 12, 24, and 48 h post-infection cells were lysed with water, lysates were serially diluted in Hank's Balanced Salt Solution and used to infect HeLa cells in 96 well plates. At 24 h post-infection, monolayers were fixed in methanol and stained with rabbit anti-EB antibody followed by anti-rabbit DyLight 488 (Jackson ImmunoResearch Laboratories, Westgrove PA). The number of inclusions per field of view were counted in 20 fields for each sample using a Leica DMI600B fluorescent microscope. Total Infectious Forming Units (IFUs)/ml were calculated for each sample. Titers for both the *C. trachomatis* L2-wild type and L2-ΔCT228 were repeated 3 times to ensure 5 μL contained 1 × 10^6^ EBs.

### Enumeration of extrusions

Extrusions were enumerated by a method similar to that described by Lutter et al. (2013). HeLa cell monolayers (50% confluence) were plated into 24-well plates and incubated overnight with MYPT1 or Scramble Targetplus Smartpool siRNA (Dharmacon, Lafayette, CO.), complexed with DharmaFECT1 in RPMI with 5% FBS. At 48 h post-transfection, cells were infected in triplicate with *C. trachomatis* L2-wild type and L2-ΔCT228 at a MOI of ~0.5. At 48 h post-infection, the supernatants were removed and gently pelleted by centrifugation at 300 x g with the resulting pellet gently resuspended in 50 μL of media. Trypan blue (ThermoFisher Scientific) and NucBlue Hoechst live-cell stain (ThermoFisher Scientific) were added and 10 μL samples were loaded onto a Hausser Bright-line hemacytometer and a total of 16 grids were counted for each condition. Extrusions free of host cell nuclei (to distinguish from an infected cell) were enumerated on a Leica DMI6000B fluorescent microscope. The entire experiment was repeated on 3 separate days to ensure consistent data across multiple trials.

### Immunofluorescence to measure MYPT1 and myosin recruitment

*C. trachomatis* L2-wild type and L2-ΔCT228 were used to infect HeLa cell monolayers on glass coverslips in 24 well plates (CellTreat Scientific, Pepperell MA) at a MOI of ~0.5 (performed in technical triplicates). At 18 h post-infection supernatants were removed and cells were fixed in cold methanol. Recruitment of host proteins was tested with primary antibody staining to MYPT1 (United States Biological Life Sciences, Salem, MA), phospho (pSer-19)-MLC2 (Abcam, Cambridge, MA), phospho (pTyr419) Src (Millipore Sigma, Burlington, MA), Myosin IIa (ThermoFisher Scientific), Myosin IIb (ThermoFisher Scientific), and phospho (pTyr 471) Myosin Light Chain Kinase (Santa Cruz). *Chlamydiae* were detected with anti-*Chlamydia* LPS (ThermoFisher Scientific). Fluorescent secondary antibodies, anti-mouse or anti-rabbit DyLight 594 and DyLight 488 (Jackson ImmunoResearch Laboratories, West Grove, PA) were used for indirect immunofluorescence. The entire experiment was repeated on three separate occasions (*n* = 3 biological replicates). Twelve images were captured per condition using a Leica DMI600B fluorescent microscope and a representative image was selected for presentation.

### Western blotting

HeLa cells were infected with *C. trachomatis* L2-wild type and L2-ΔCT228; at different time points during infection, cells were lysed with 2X Laemmli Sample Buffer (Biorad, Hercules, CA). Protein samples were separated by SDS-PAGE electrophoresis (12 or 15% gel) and transferred to 0.2 um nitrocellulose membrane (Bio-Rad, Hercules, CA). Membranes were blocked with 5% non-fat dry milk/5% BSA in 1X Tris-buffered saline containing 0.1% Tween-20 (TBST) for 1 h at room temperature. After blocking, membranes were incubated with primary antibodies diluted in 5% non-fat milk/5% BSA in 1X TBST at 4°C overnight followed by incubation with horseradish peroxidase conjugated anti-rabbit/mouse antibodies (Cell Signaling, Danvers, MA) for 1 h at room temperature. Detection was performed by enhanced chemiluminescence using SignalFire ECL reagents (Cell Signaling Technology, Danvers, MA). Immunoblot images were acquired using the Fluorchem E FE0622 system (ProteinSimple, San Jose, CA). Primary antibodies to MLC2 (Millipore Sigma, Burlington, MA), p-MLC2 (S19) (ThermoFisher Scientific), HsP60 (ThermoFisher Scientific), GAPDH (Santa Cruz), and MYTP1 (Cell Signaling, Danvers, MA) were used herein.

### Intravaginal infection of mice

Six-week old female inbred C3H/HeJ mice were purchased from Jackson Laboratories (Bar Harbor, Maine). All animal work was performed according to The Animal Care and Use Guidelines and approved by the Institution of Animal Care and Use Committee at Oklahoma State University. At 7 and 3 days prior to infection, all mice were subcutaneously injected with a depot formulation of 2.5 mg of medroxyprogesterone acetate (Upjohn, Kalamazoo, MI) to synchronize estrous prior to infection. Mice were infected intravaginally with 1 × 10^6^ *C. trachomatis* L2-wild type EBs or L2-ΔCT228 EBs in 5 μL sucrose-phosphate-glutamate (SPG) to monitor both the course of infection and immune response (*n* = 8 infected/colonized with L2-wild type; *n* = 9 infected/colonized with L2-ΔCT228) and histopathology (*n* = 5 for L2-wild type and L2-ΔCT228). Due to the inherent technical imperfection of the intra vaginal route of infection using small volumes (e.g., 5 μL) and that the human L2 serovar produces less robust colonization relative to other strains, any mice swabbed early (Day 7) post-infection who shed 0 IFU (100% clear of infection) were not processed any further.

### Quantification of recoverable IFUs from the cervicovaginal tract

At days 7, 14, 21, 28, and 42 days post-infection, cervicovaginal tracts were swabbed (Puritan Diagnostics, HydraFlock 6″ 15 cm swabs; Guilford, ME) and each swab was added to tubes containing 600 μL SPG and two glass beads on ice, as adopted from previously described studies (Shaw et al., 2001, 2017). Swab samples were vortexed vigorously to liberate EBs from the swab followed by serial dilution and inoculation of confluent HeLa cell monolayers in 96 well plates (CellTreat Scientific, Pepperell MA). Plates were centrifuged at 700 × g for 1 h to promote EB entry, then incubated at 37°C for 30 min. Inoculated HeLa monolayers were washed to remove any extracellular EBs then incubated in RPMI 1640 + 5% FBS + gentamicin (10 μg/mL) for 24 h at 37°C with 5% CO_2_. Plates were fixed with methanol and stained with anti-MOMP (courtesy of Dr. Harlan Caldwell) followed by anti-mouse DyLight 488 (Jackson ImmunoResearch). Twenty fields of view were counted using a Leica MI6000B fluorescent microscope. Total recoverable IFUs per mouse were calculated for each sample. While variation ranging across 2 logs of recoverable IFU exists throughout the *Chlamydia* murine model literature and periodically within the same study, replicated studies using this current L2 stock at 1 × 10^6^ per mouse consistently produces 10^4^-10^5^ recoverable IFU at early time points post-murine infection and is therefore used for internal comparison to mice infected with 1 × 10^6^ L2-ΔCT228 EBs herein.

### Quantification of systemic and mucosal antibody response

To measure systemic and mucosal immune responses to infection, sera and vaginal lavages were collected 31 days post-infection for ELISA. Vaginal lavages were obtained by gently washing the vaginal vault with 60 μL 0.5% BSA-PBS twice and stored at −20°C until the ELISA was performed. Briefly, 96-well polystyrene plates (Immulon 2HB; Thermo, Milford, MA) were coated with 1 μg of formalin fixed EBs (serovar L2) per well in 100 μL TBS (50 mM Tris buffer pH 7.5, 0.15 M NaCl) overnight at 4°C. Following adsorption of fixed EBs, wells were washed to remove unbound EBs then blocked with 200 μL 2% BSA in 0.012 M Tris pH 7.4, 0.14 M NaCl, 3.0 mM KCl, 0.05% Tween 20 for 90 min at 37°C. Blocking solution was removed and wells were washed, then serial dilutions of sera and vaginal washes were added and incubated for 90 min at 37°C. *Chlamydia*-specific antibody titers were measured using alkaline phosphatase-conjugated anti-mouse IgG2a (sera samples) and anti-mouse IgG and IgA antibodies (vaginal lavage samples) (Southern Biotech Associates, Birmingham, AL). P-nitrophenyl phosphate (pNPP) was used as a substrate and resulting absorbance was read at 405 nm (BioTek Synergy, Winooski, VT). Antibody titers were considered positive at the highest sample dilution that displayed an absorbance that was ≥3X the absorbance of concentrated sham (uninfected) samples.

### Reproductive tract histology

Mouse uteri were excised and immersion fixed in 10% buffered neutral formalin on days 23 and 64 post-infection for scoring inflammatory damage. Following fixation, they were processed in whole and embedded *en bloc* into paraffin followed by sections cut at 4 μm and stained with hematoxylin and eosin (H&E). Sections were examined and scored via light microscopy by an American College of Veterinary Pathologists (ACVP) certified veterinary pathologist with the following numerical designations: 0, normal; 1, minimal change; 2, mild change; 3, moderate change; 4, severe change. Scored parameters (mean ± SE) included an overall impression, periglandular mucinous change, hydrosalpinx, uterine tubal dilation, and luminal PMN. Morphometric analyses of endometrial luminal epithelial height were determined using calibrated measurements via Olympus CellSens software coupled to an Olympus DP70 microscope camera. Each reproductive tract was evaluated regionally and morphometrically at six locations (proximal, mid-, and distal locations of the two uterine horns) determined to be uniform and free of tissue bends and curves.

### Statistics

Student's unpaired two-tailed *t*-test with equal variance was used to compare mean ± SE growth (IFUs) and extrusion numbers between L2-wild type and L2-ΔCT228. One-way ANOVA repeated measures was used to analyze the recoverable log IFU data across four time points following murine infection with L2-wild type or L2-ΔCT228. Student's unpaired two-tailed *t*-test was used to analyze systemic (IgG2a) and mucosal (IgA, IgG) mean ± SE antibody titers in mice infected with L2-wild type vs. L2-ΔCT228 and performed on clinical scores for histopathology between L2-wild type and L2-ΔCT228 infected mice. Statistical analyses were performed using Prism 5.0 and data were considered significant at p < 0.05.

## Results

### *C. trachomatis* CT228 deletion and resulting effects

**Figure 1:**
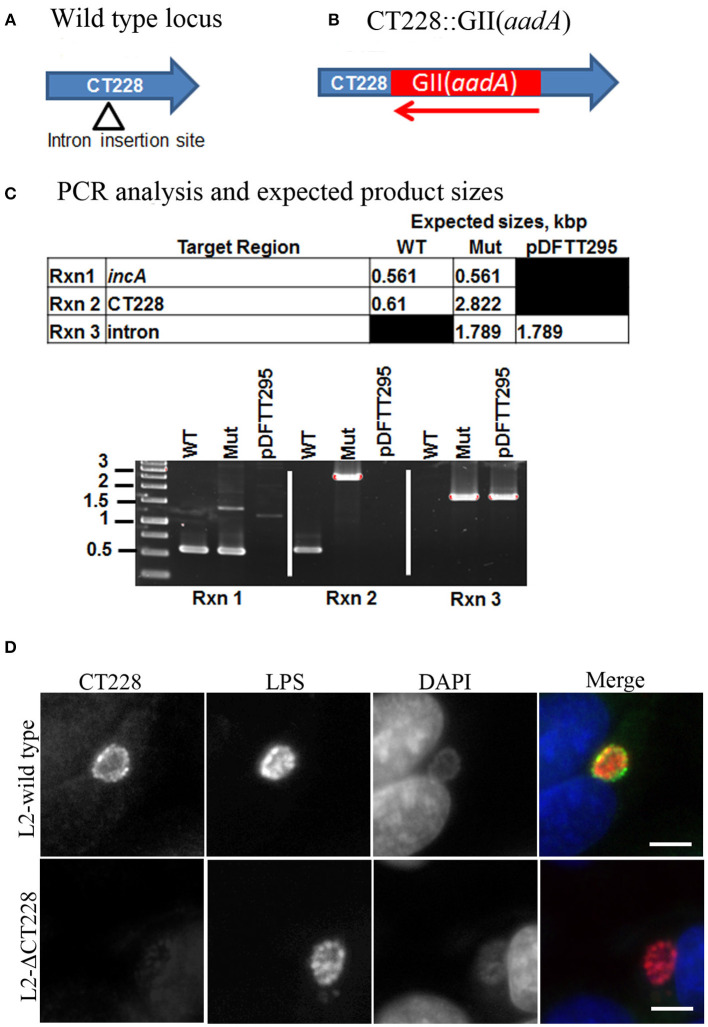
TargeTron inactivation of *CT228*. **(A)** TargeTron insertion site in *CT228*. **(B)** Schematic of *CT228* containing GII(*aadA*). **(C)** PCR verification of TargeTron insertion in L2-ΔCT228. PCRs were used to amplify *incA, CT228* and the Intron in the L2-wild type, L2-ΔCT228 (Mut), and TargeTron vector (pDFTT295). **(D)** Immunofluorescence images of L2-wild type and L2-ΔCT228 at 18 h post-infection stained with anti-CT228 and anti-*Chlamydia* LPS followed by fluorescent secondary antibodies. Scale bar, 10 μm.

The chlamydial TargeTron vector pDFTT3(bla) was modified to allow for spectinomycin selection by replacing the *bla* cassette with the *aadA* cassette. The vector was then retargeted to insert into *CT228* as previously described (Johnson and Fisher, 2013). The predicted insertion site was at bp 295 within *CT228* in an antisense orientation. Mutants were selected using 500 μg/ml spectinomycin and plaque purified to obtain a clonal L2-ΔCT228 strain. The *CT228* locus map, TargeTron insertion site, and PCR validation of the intron insertion are shown in Figures 1A–C. Sequencing of the L2-ΔCT228 mutant CT228::GII(*aadA*) locus confirmed intron insertion at the predicted location. HeLa cell monolayers were infected with both L2-wild type and L2-ΔCT228 for 18 h and processed for immunofluorescence using anti-CT228 and anti-LPS antibodies. CT228 is detected surrounding the chlamydial inclusion in L2-wildtype, however is absent in the L2-ΔCT228 inclusion membrane confirming the inactivation of *CT228* (Figure 1D). Southern blotting confirmed a single TargeTron insertion in the L2-ΔCT228 mutant (Figure S1).

**Figure 2:**
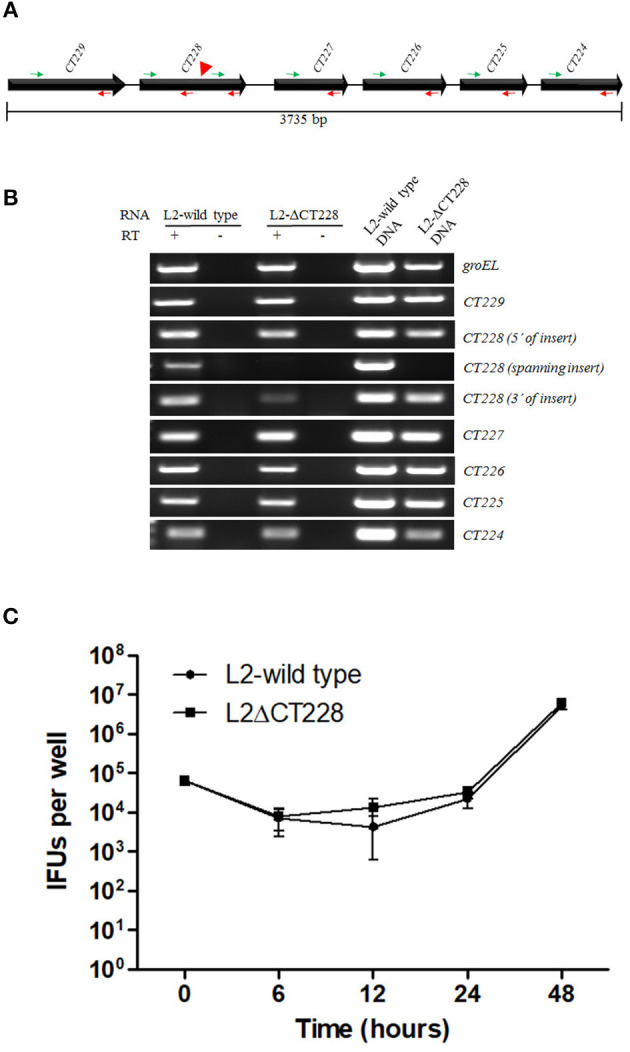
TargetTron inactivation of *Chlamydia trachomatis CT228* and subsequent *CT229-CT224* gene linkage group transcription. **(A)** Physical map of *C. trachomatis CT229-CT224* gene linkage group. Solid arrows represent *CT229-CT224* ORFs. Gene designations are indicated above each ORF. Green and red arrows indicate forward and reverse primers, respectively, used for RT-PCR analysis. Red arrowhead indicates the point of TargeTron insertion within *CT228*. **(B)** Agarose gel image(s) show RT-PCR products corresponding to *groEL* (control), and *CT229-CT224* gene linkage group expression (correlating to the indicated primers above). Total RNA (L2-wild type or L2-ΔCT228) is indicated above corresponding gel lanes. + indicates RT added to reaction. – Indicates RT not added to reaction. L2-wild type and L2-ΔCT228 DNA act as PCR controls. **(C)** Growth curves of L2-wild type and L2-ΔCT228 were performed in triplicate, the entire experiment was repeated on three separate occasions and a representative growth curve was selected. Error bars represent standard deviation.

The *CT229* through *CT224* gene linkage map suggests that these genes are transcriptionally linked as an operon (Stephens et al., 1998). In effort to determine whether the TargeTron insertion in *CT228* may exert polar effects on the transcription of other genes within the putative operon, RT-PCR analysis was performed using oligonucleotide primers (Table S1) designed within each ORF as well as spanning the TargeTron insertion site (Figure 2A). The green and red arrows in Figure 2A indicate the position of forward and reverse primers, respectively. Using total RNA harvested from Hela cells infected with *C. trachomatis* L2-wild type as template, amplification products were observed (Figure 2B) for each ORF (*CT229, CT228, CT227, CT226, CT225*, and *CT224)*. In total RNA from *C. trachomatis* L2-ΔCT228 infected cells, robust amplification products were observed (Figure 2B) for *CT229, CT227, CT226, CT225*, and *CT224*. Of particular note is the observation that the region of *CT228* that lies upstream of the TargeTron insertion also produced a robust amplification product, while primers spanning the insertion site do not. It was also observed that RT-PCR using primers situated downstream of the TargeTron insertion site, yet within the *CT228* ORF, produce a far less robust amplification product (Figure 2B). Taken together, the data indicate that the TargeTron insertion within *CT228* does not cause a polar elimination of *CT227, CT226, CT225, and CT224* transcripts while it does interrupt the production of a complete *CT228* transcript.

### Comparison of bacterial growth, recruitment of MYPT1, and extrusion production in *C. trachomatis* L2-wild type vs. L2-ΔCT228

**Figure 3:**
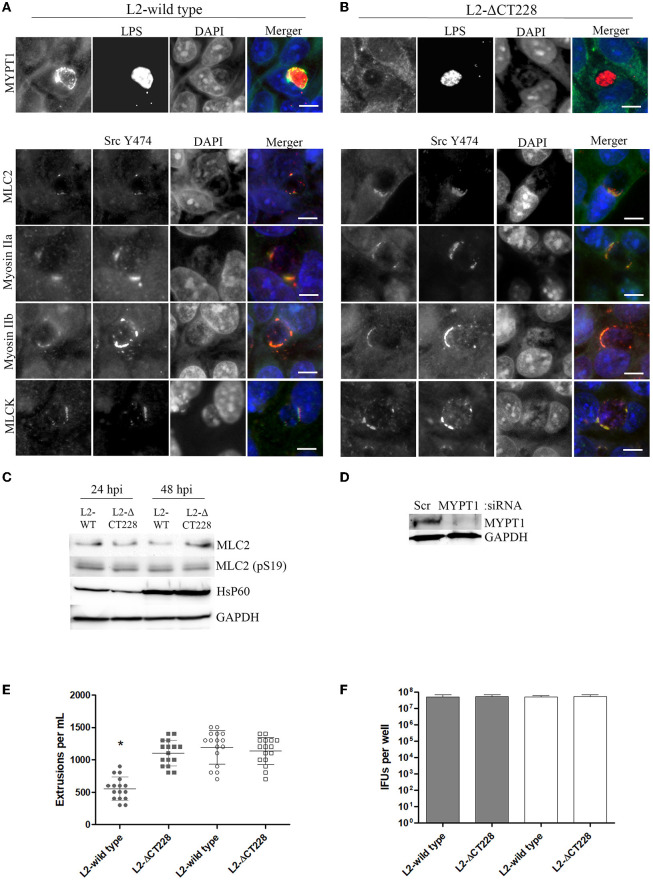
Recruitment of MYPT1 and Myosin phosphatase pathway components and extrusion production by *C. trachomatis* L2-wild type and L2-ΔCT228. HeLa cell monolayers were infected at a MOI of ~0.5 with L2-wild type and L2-ΔCT228 for 18 h (in technical triplicate). Cells were fixed and stained with primary antibodies to MYPT1, *Chlamydia* LPS, MLC2 (pS19), Src Y474, MLCK (pY471), non-muscle Myosin IIa and IIb followed by fluorescent secondary antibodies. Experiments were repeated on three separate occasions and representative images were selected. **(A,B)** Top panel shows individual and merged images of MYPT1 recruitment (green) and *Chlamydia* LPS staining (red) in both the L2-wild type and L2-ΔCT228. Lower panel of individual and merged images show MLC2 (pS19), MLCK (pY471), and Mysoin IIa and IIb (green) co-localizing with active Src Y474 kinase (red) in microdomains at the periphery of inclusions in both L2-wild type and L2-ΔCT228. Scale bar, 10 μm. **(C)** Total protein from L2-wild type and L2-ΔCT228 infected HeLa cells at 24 and 48 h post-infection were assessed for MLC2, MLC2 (pS19), HsP60, and GAPDH levels by western blot analysis. **(D)** HeLa cells were treated with either Scramble (Scr) or MYPT1 siRNA for 48 h prior to infection with L2-wild type and L2-ΔCT228. Protein samples were assessed for MYPT1 and GAPDH levels by western blot. **(E)** Extrusions collected and **(F)** IFUs were assessed for L2 wild-type and L2-ΔCT228 at 48 h post-infection in either Scramble (symbols and solid bars) or MYPT1 (open symbols and white bars) siRNA treated HeLa cells. ^*^p < 0.0001.

Bacterial growth was measured at 0, 6, 12, 24, and 48 h post-infection of HeLa cell monolayers with *C. trachomatis* L2-wild type and L2-ΔCT228. There were no statistically significant differences between growth curves of the different strains and the curves followed the expected pattern for growth (Figure 2C). L2-wild type and L2-ΔCT228 were assessed for recruitment of MYPT1 and myosin phosphatase pathway host proteins at 18 h post-infection in HeLa cell monolayers. While the L2-wild type strain recruited MYPT1 to the periphery of the inclusion (Figure 3A), the L2-ΔCT228 failed to do so (Figure 3B). However, both strains demonstrate recruitment of MLCK (pY474), MLC2 (pS19), and Myosin IIa and Myosin IIb to the inclusion co-localizing with active Src Y474 in the microdomains at 18 h post-infection (Figures 3A,B). Recruitment of MLCK (pY474), MLC2 (pS19), and Myosin IIa and Myosin IIb at 48 h post-infection was also visualized confirming recruitment for the duration of the infection (Figure S2). Western blot analysis of MLC2 (pS19) and endogenous MLC2 levels did not show any differences during the course of infection at 24 and 48 h post-infection (Figure 3D). The number of extrusions produced by both L2-wild type and L2-ΔCT228 was quantified at 48 h post-infection in Scramble or MYPT1 siRNA depleted HeLa cells [successful MYPT1 knockdown was verified by western blot (Figure 3E)]. In the Scramble treated cells, L2-ΔCT228 produced significantly more extrusions than the L2-wild type (Figure 3E, *p* < 0.0001) however, there was no difference in extrusion production between the L2-wild type and L2-ΔCT228 in the MYTP1 siRNA treated cells (Figure 3E). Analysis of total *Chlamydia* IFUs was comparable between strains and conditions (Figure 3F).

### Course of *C. trachomatis* L2-wild type vs. L2-ΔCT228 infection *in vivo*

**Figure 4:**
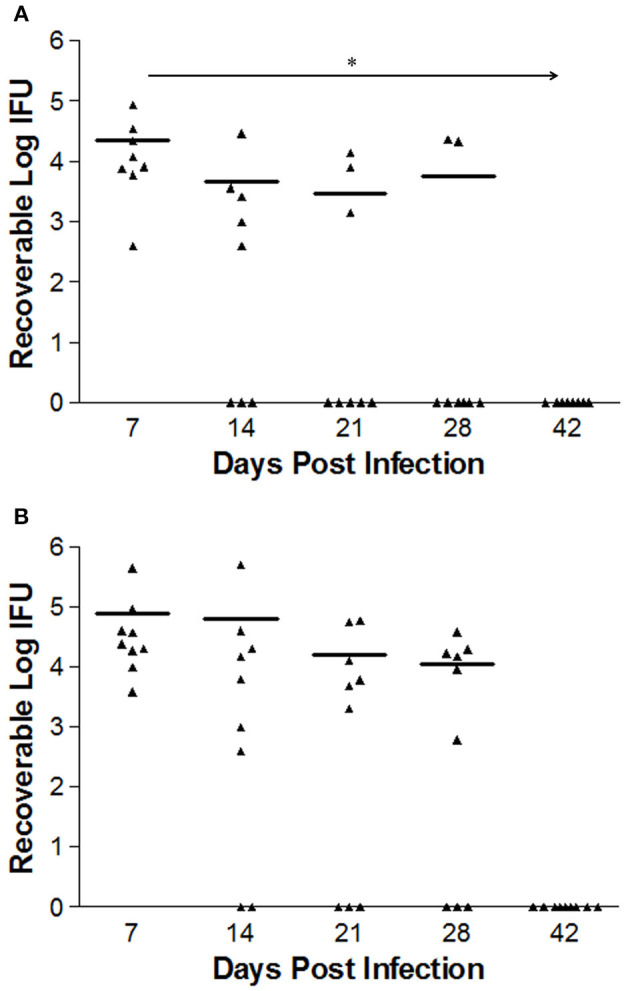
Recoverable IFUs shed by mice infected with *C. trachomatis* L2-wild type and L2-ΔCT228. Female C3H/HeJ mice were intravaginally infected with 1 × 10^6^ EBs of either L2-wild type or L2-ΔCT228. Recoverable IFUs were obtained by swabbing vaginal tracts and enumerating on HeLa cell monolayers. Recoverable IFU data are expressed for **(A)** L2-wild type (*n* = 8) and **(B)** L2-ΔCT228 (*n* = 9) on a logarithmic scale from Day 7 to 42 post-infection. Effect of time (^*^p = 0.006) was observed in mice infected with L2-wild type (repeated measures one-way ANOVA). For day 28, 6/8 L2-wild type infected mice were clear compared to 3/9 L2- ΔCT228. Triangles represent individual mice, bars represent mean of group for each time point.

Bacterial burden in mice infected intravaginally with either 1 × 10^6^ L2-wild type EBs (*n* = 8) or L2-ΔCT228 EBs (*n* = 9) was quantitated at days 7, 14, 21, and 28 via swabbing the cervicovaginal tract and culturing on HeLa cell monolayers. The calculated numbers of recoverable log IFUs are shown in Figure 4. Early L2-wild type (Figure 4A) and L2-ΔCT228 (Figure 4B) infection in mice at day 7 produced an infectious burden that was not statistically different from one another. However, there was a statistically significant effect of time on the clearance of L2-wild type (*p* = 0.006) that was not present in response to L2-ΔCT228 (*p* = 0.371).

### Systemic and mucosal antibody response to *C. trachomatis* L2-wild type vs. L2-ΔCT228

**Figure 5:**
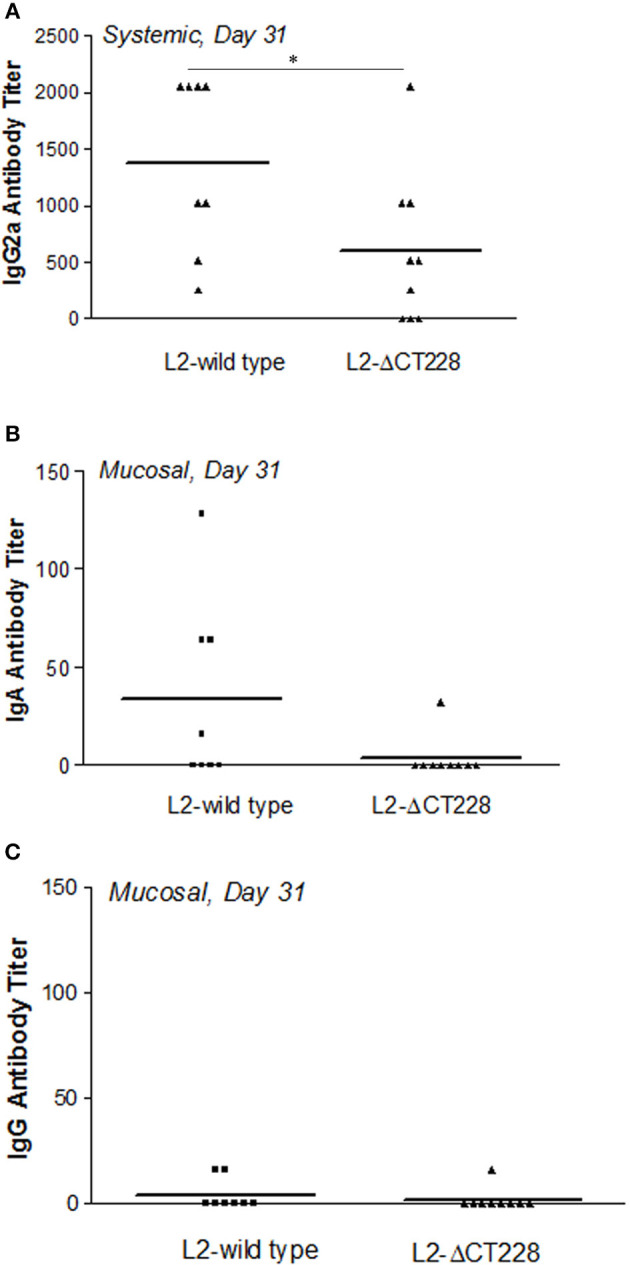
Systemic and mucosal antibody titers following infection with *C. trachomatis* L2-wild type and L2-ΔCT228. **(A)** Sera were collected from mice 31 days post-infection with L2-wild type (*n* = 8) or L2-ΔCT228 (*n* = 9) and assayed for the presence of anti-chlamydial IgG2a. **(B,C)** Vaginal lavages were collected 31 days post-infection and assayed for the presence of anti-chlamydial IgA and IgG. Antibody titers are expressed for individual mice as the highest dilution tested that produced >3-fold the absorbance as control/uninfected mice. Bars represent mean antibody titer per group. ^*^p = 0.0412, unpaired two-tailed Student *t*-test.

Mean systemic anti-chlamydia IgG2a antibody titers were significantly higher (*p* = 0.041) in mouse sera at day 31 post-infection with L2-wild type (*n* = 8) relative to L2-ΔCT228 (*n* = 9) as shown in Figure 5A. As expected for serovar L2, mucosal anti-*Chlamydia* antibody responses were low overall. There were negligible mucosal anti-*Chlamydia* IgG and IgA titers in mice 31 days post-infection with L2-ΔCT228 relative to L2-wild type (Figures 5B,C).

### Histopathology in *C. trachomatis* L2-wild type vs. L2-ΔCT228 infected mice

**Figure 6:**
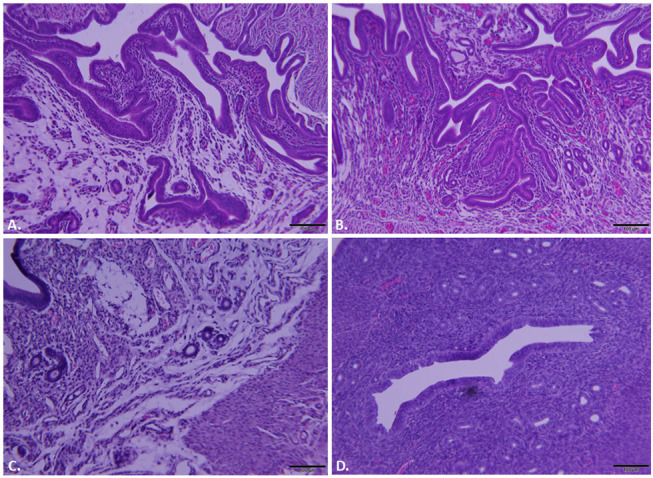
Histopathological assessment of reproductive tracts post-infection. Mice (*n* = 5 per group) were euthanized at 23 and 64 days post-infection (dpi) and entire reproductive tracts were removed and formalin fixed for histology. Representative images of H&E stained sections of uterine tissue were captured using an Olympus DP70 for mice infected with **(A)** *C. trachomatis* L2-wild type, 23 dpi, **(B)** *C. trachomatis* L2-ΔCT228, 23 dpi, **(C)** *C. trachomatis* L2-wild type, 64 dpi, **(D)** *C. trachomatis* L2-ΔCT228, 64 dpi. See Table 1 for pathological clinical scoring of reproductive tracts at 64 dpi.

Whole reproductive tracts were collected 23 and 64 days post-infection (L2-wild type vs. L2-ΔCT228) and formalin fixed for histological analyses. Sections of uterine tissue were examined and the scores assigned by an AVCP certified veterinary pathologist via light microscopy. Individual clinical scores ranged from normal to moderate pathology for the infected mice (Table 1, raw scores provided in Table S2). As previously shown, intravaginal infection with serovar L2 overall produces less histopathology relative to other human serovars (e.g., serovar D-LC) and the mouse-specific pathogen *C. muridarum* (MoPn) (Shaw et al., 2017). A single infection with serovar L2 produces only minimal to mild histopathology, however it was important to assess any inflammatory damage following infection with L2-ΔCT228 since this strain had never previously been tested *in vivo* and studies support the concept that chlamydial genotypes may alter the capacity for ascension and survival within the upper genital tract (Menon et al., 2015). Histological parameters compared between L2-wild type and L2-ΔCT228 revealed no statistical differences with the exception of fewer mucinous changes observed in the L2-ΔCT228 infected mice (Figures 6A–D and Table 1, *p* = 0.02).

**Table 1:** Pathological scoring of murine reproductive tracts.

**Infectious agent**	**Overall impression**	**Mucinous change**	**Hydrosalpinx,**	**Uterine tubal dilation**	**Uterine luminal PMN**	**Representative images**
*L2-Wild type*	1.0 (±0.55)	1.4 (±0.24)	0	1.4 (±0.51)	0	Figures 6A,C
*L2-ΔCT228*	0.4 (±0.40)	0.4 (±0.24)^*^	0	0.6 (±0.40)	0	Figures 6B,D

*Histological sections of reproductive tracts (n = 5/group at 64 dpi) were examined and scored by an ACVP certified veterinary pathologist via light microscopy with the following numerical designations: 0, normal; 1, minimal change; 2, mild change; 3, moderate change; 4 severe change (mean ± SE).*

* *p = 0.02 (L2-wild type vs. L2-ΔCT228). For raw scores, please see Table S2*.

## Discussion

The nexus of host-pathogen interaction during chlamydial infection resides at the inclusion membrane. It is at this interface that *Chlamydia* exploit host cellular machinery to scavenge materials necessary to build and maintain their intracellular niche. Inc proteins are characterized by a bilobed hydrophobic domain of ~40 amino acids and are well conserved between *C. trachomatis* serovars implying a potential role in pathogenesis (Weber et al., 2015). While there are as many as 59 predicted Incs (Bannantine et al., 2000; Toh et al., 2003; Dehoux et al., 2011; Lutter et al., 2012), the host-pathogen functional interaction of relatively few is currently understood. The most extensively studied is IncA (CT119), which encodes SNARE-like motifs and is required for the homotypic fusion of inclusions that occurs late in infection (Hackstadt et al., 1999; Delevoye et al., 2008). IncB (CT232) is localized to microdomain regions saturated with Src kinases that participate in pathways governing inclusion trafficking and intracellular growth (Mital et al., 2010; Mital and Hackstadt, 2011). IncD (CT115) recruits ceramide endoplasmic reticulum transferase (CERT) (Agaisse and Derre, 2014), a lipid transfer protein, while IncG (CT118) recruits adaptor protein 14-3-3β to enhance host cell viability (Scidmore and Hackstadt, 2001). Endosomal trafficking is regulated by interaction between CT229 and GTPase Rab4 (Rzomp et al., 2006), CT850 interaction with dynein drives migration to the MTOC (Mital et al., 2015), IncV (CT005) interacts with the endoplasmic reticulum (ER) integral membrane protein VAP securing the chlamydial inclusion to the ER (Stanhope et al., 2017)and CT813 binding with ARF1 and ARF4 bring inclusions in close proximity to Golgi mini-stacks (Wesolowski et al., 2017). Later during infection, both CT101 and CT228 regulate host cell exit via extrusion; CT101 (also known as MrcA) interacts with host inositol tri-phosphate (IP_3_) for calcium management (Nguyen et al., 2018) and, as previously mentioned, CT228 recruits myosin phosphatase (MYPT1) to the inclusion membrane (Lutter et al., 2013; Mirrashidi et al., 2015).

Whether *Chlamydia* exit host cells via lysis or extrusion is controlled by the phosphorylation state of MLC2. Dephosphorylated MLC2 favors lysis while phosphorylated MLC2 enhances extrusion (Lutter et al., 2013); the extent of phosphorylation is determined by the ongoing opposing activities of myosin phosphatase (MYPT1) and myosin kinase (MLCK). In this study we generated a targeted chromosomal mutation of *CT228* using the TargeTron system (Johnson and Fisher, 2013) that no longer produced CT228 (Figure 1D). Gene interruption by insertion mutagenesis introduces the possibility of generating polar effects on downstream gene expression. This can confound subsequent findings as to which genes are specifically responsible for an observed effect. We used RT-PCR to analyze total RNA from L2-wild type and L2-ΔCT228 infected Hela cells. The data confirms that L2-ΔCT228 does not produce a full-length *CT228* transcript during infection (Figure 2B). However, it does produce transcripts for *CT227, CT226, CT225*, and *CT224*, all of which lie downstream of *CT228* and the TargeTron insertion (Figures 2A,B). Analysis of the intervening sequence (282 bp) upstream of the *CT227* start site revealed −35 and −10 sigma factor binding sequences (TTGTGT-17bp-TATTAT) with strong homology to the Chlamydial σ^66^ (Yu et al., 2006) consensus binding sequences (TTGACA-17bp-TATTAT), suggesting that a promotor exists for transcription of *CT227-CT224* which may be in addition to upstream promotors. These findings indicate that for this putative operon, the insertion does not eliminate the production of downstream gene products, focusing our findings on CT228 cellular interactions.

A direct comparison of the L2-wild type and L2-ΔCT228 *in vitro* demonstrated that the only identified alteration in protein localization exhibited by cells infected with the CT228 mutant was in the recruitment of MYPT1 to the inclusion (Figure 3B), confirming the role of CT228. The remaining components of the myosin pathway (MLCK, MLC2, and heavy chains Myosin IIa and IIb) showed identical recruitment by L2-wild type and L2-ΔCT228 (Figures 3A,B; Figure S2) suggesting that myosin regulated events such as extrusion ought to be intact and theoretically enhanced in the mutant. If the recruitment of MYPT1 to the chlamydial inclusion is removed (thus loss of phosphatase activity) and MLCK, which phosphorylates and activates MLC2, continues to be recruited then we would predict a two-pronged increase in phosphorylated MLC2 that is localized to the inclusion and subsequent increase in extrusion production. Our observation that L2-ΔCT228 produced significantly more extrusions compared to the L2-wild type supports this hypothesis (Figure 3). This further suggests that while CT228 is not required for extrusion production in *Chlamydia*, it likely influences the rate of extrusions and may have implications *in vivo*. The notion that CT228 is non-essential for extrusion production is not novel. Previous studies utilizing the ocular isolate B/Jali20/OT, which contains a natural truncation mutation of CT228 among other SNPs in its genome, have reported a lack of MYPT1 recruitment (Lutter et al., 2013) and, interestingly, a decrease in extrusion production compared to serovar L2 (Zuck et al., 2016b). Although the extrusion mechanism is conserved among Chlamydiae, a comparison of extrusion production by different species or non-isogenic strains carrying multiple mutations such as B/Jali20/OT may confound efforts to verify roles for specific genes. The cross serovar comparison of extrusion production could account for the reported decrease in extrusions observed in B/Jali20/OT, whereas in our study using isogenic strains we observe a significant increase in extrusions produced by L2-ΔCT228 when compared to the L2-wild type.

Considering our finding that *in vitro* growth of L2-ΔCT228 produced significantly more extrusions than L2-wild type, we hypothesized that this may provide a means to safeguard *Chlamydia* from the host immune response *in vivo*. While both extrusion and lysis occur late infection, the favoring toward even a minor increase in extrusion may dampen the ability of the host immune response to recognize EBs due to enclosure within extrusions, which are surrounded by host membrane. Enhancement of host egress via extrusion would theoretically reduce the number of opportunities for presentation of EB antigens and may explain the modest decrease in clearance of mice infected with L2-ΔCT228. Our data show that murine intravaginal infection with L2-ΔCT228 cleared more slowly; only 3/9 L2-ΔCT228 infected mice were clear by day 28 compared to 6/8 L2-wild type infected mice (Figures 4A,B). Likewise, significantly lower systemic anti-*Chlamydia* IgG2a titers were produced by L2-ΔCT228 infected mice relative to L2-wild type at day 31 post-infection (Figure 5A). As expected in response to serovar L2 infection, there were negligible mucosal IgG and IgA titers in both groups 31 days post-infection (Figures 5B,C). Moreover, histological analyses of whole murine reproductive tissue show a significant, albeit minor, decrease in mucinous changes following infection with L2-ΔCT228 compared to L2-wild type within this current study (Figures 6A–D). It is important to note that since serovar L2 is well known to elicit only minimal histopathological changes, interpretation of these findings is restricted by the compression of the 0–4 clinical scoring scale to 0–2. Clinical scores of mucinous changes from our previous studies in L2-infected mice displayed a somewhat lower but overlapping numerical range for mucinous changes despite higher recoverable IFU (Shaw et al., 2017) than that which was observed in this current study confounding any definitive conclusions as to the physiological relevance of and reliance on this particular parameter between separate studies. Overall, our *in vivo* findings suggest a delay in clearance following intravaginal infection with L2-ΔCT228 with a concomitant reduction in anti-*Chlamydia* humoral responses. Taken together with evidence that the mutant and wild-type strains exhibit similar growth, we speculate that the difference in EB release (via lysis or extrusion) may alter the degree of recognition by macrophages and dendritic cells thereby affecting the ability of host immune clearance mechanisms. While there are multiple players involved in clearance, the data suggest that loss of MYPT1 recruitment (as a result of CT228 disruption) affects the longevity of infection *in vivo*, which may be related to the degree of host cell exit via extrusion.

## Future directions

It will be important to elucidate the mechanism whereby this chlamydial mutant evades and/or alters the immune response. Future studies on how CT228 may specifically affect the required events for antigen presentation and development of protective immunity, such as dendritic cell activation, IFN-γ production, CD4 T cells mucosal imprinting as well as antibody-mediated cytotoxic (neutrophil) events, are warranted and may reveal survival strategies available to *Chlamydia* that could also serve as targets for new therapies. Generating a CT228 mutant in serovars that are more robust in driving histopathology to better model human disease than L2 is crucial. Likewise, it would be of interest to investigate whether the loss of CT228 protein affects the localization and function of other Incs.

## Author contributions

EL: project conceptualization, data acquisition, data analysis, data interpretation, revision of manuscript, principle investigator; JS: project conceptualization, data acquisition, data analysis, data interpretation, revision of manuscript, co-principal investigator; DF: construction of L2-ΔCT228, revision of manuscript; TS: data acquisition, data analysis; CK: data acquisition, data interpretation, revision of manuscript; ES: data acquisition, data interpretation, revision of manuscript; PS: performed all protein and western blotting experiments.

### Conflict of interest statement

The authors declare that the research was conducted in the absence of any commercial or financial relationships that could be construed as a potential conflict of interest.
