# Corrigendum: Genetic inactivation of *Chlamydia trachomatis* inclusion membrane protein CT228 alters MYPT1 recruitment, extrusion production, and longevity of infection

**DOI:** 10.3389/fcimb.2022.1075768

**Published:** 2023-02-14

**Authors:** Jennifer H. Shaw, Charlotte E. Key, Timothy A. Snider, Prakash Sah, Edward I. Shaw, Derek J. Fisher, Erika I. Lutter

**Affiliations:** ^1^Department of Integrative Biology, Oklahoma State University, Stillwater, OK, United States; ^2^Department of Veterinary Pathobiology, Oklahoma State University, Stillwater, OK, United States; ^3^Department of Microbiology and Molecular Genetics, Oklahoma State University, Stillwater, OK, United States; ^4^Department of Microbiology, Southern Illinois University, Carbondale, IL, United States

**Keywords:** *Chlamydia*, extrusion, lymphogranuloma venereum, L2 serovar, sexually transmitted infection, urogenital infection, CT228, mouse infection

## Error in Figures/Table

In the published article, there was an error in **Figure 3A**; panel MLC2 as published: Images for MLCK were duplicated in place of MLC2. The corrected **Figure 3A**; panel for MLC2 and its caption appear below.

**Figure 3:**
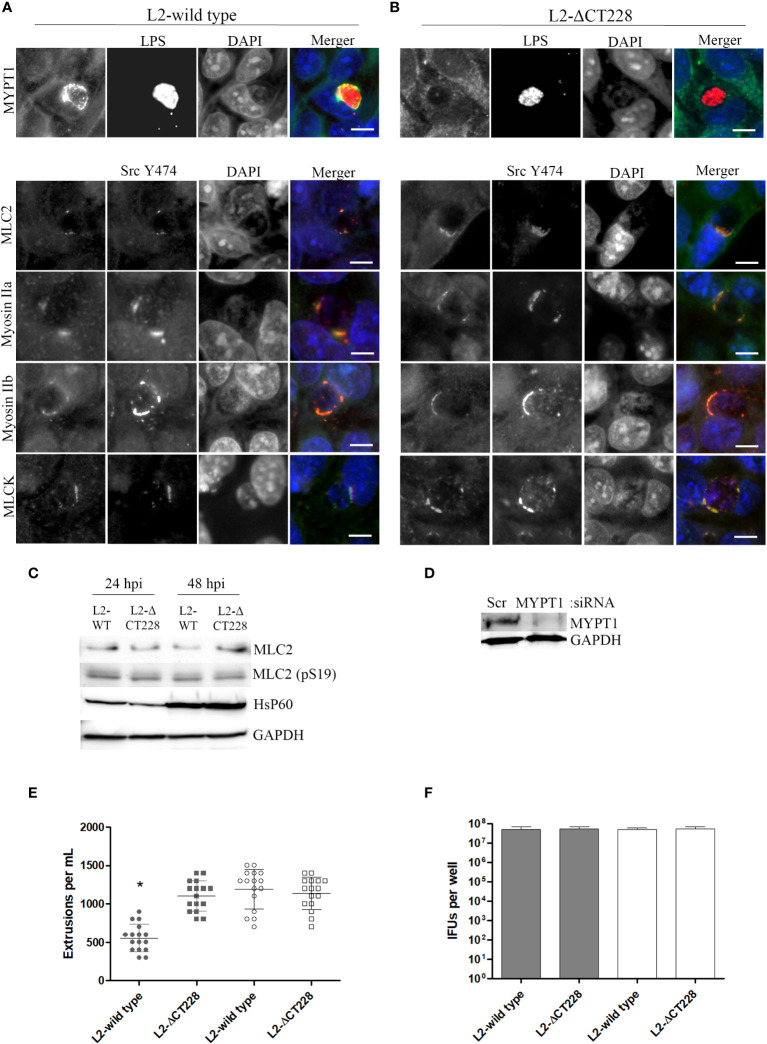
Recruitment of MYPT1 and Myosin phosphatase pathway components and extrusion production by C. trachomatis L2-wild type and L2-1CT228. HeLa cell monolayers were infected at a MOI of ∼0.5 with L2-wild type and L2-1CT228 for 18 h (in technical triplicate). Cells were fixed and stained with primary antibodies to MYPT1, Chlamydia LPS, MLC2 (pS19), Src Y474, MLCK (pY471), non-muscle Myosin IIa and IIb followed by fluorescent secondary antibodies. Experiments were repeated on three separate occasions and representative images were selected. **(A, B)** Top panel shows individual and merged images of MYPT1 recruitment (green) and Chlamydia LPS staining (red) in both the L2-wild type and L2-1CT228. Lower panel of individual and merged images show MLC2 (pS19), MLCK (pY471), and Mysoin IIa and IIb (green) co-localizing with active Src Y474 kinase (red) in microdomains at the periphery of inclusions in both L2-wild type and L2-1CT228. Scale bar, 10µm. **(C)** Total protein from L2-wild type and L2-1CT228 infected HeLa cells at 24 and 48 h post-infection were assessed for MLC2, MLC2 (pS19), HsP60, and GAPDH levels by western blot analysis. **(D)** HeLa cells were treated with either Scramble (Scr) or MYPT1 siRNA for 48 h prior to infection with L2-wild type and L2-1CT228. Protein samples were assessed for MYPT1 and GAPDH levels by western blot. **(E)** Extrusions collected and **(F)** IFUs were assessed for L2 wild-type and L2-1CT228 at 48 h post-infection in either Scramble (symbols and solid bars) or MYPT1 (open symbols and white bars) siRNA treated HeLa cells. *p < 0.0001.

The authors apologize for this error and state that this does not change the scientific conclusions of the article in any way. The original article has been updated.

