# Supplementary material for: Genetic Inactivation of Chlamydia trachomatis Inclusion Membrane Protein CT228 Alters MYPT1 Recruitment, Extrusion Production, and Longevity of Infection
Source: Front Cell Infect Microbiol. 2018 Nov 30;8:415. doi: 10.3389/fcimb.2018.00415 (PMC6284022; doi:10.3389/fcimb.2018.00415)
Supplement: Supplementary file 1 [file Table_1.DOCX]

**Table S1. Gene targets, primer sequences and methods employed to generate data shown Figure 2B.**

| ***Gene Target*** | ***Oligo Designation*** | ***Gene Primer Sequence* (5’→ 3’)** | ***Purpose*** |
| --- | --- | --- | --- |
| ***CT229*** | CT229F2^f^ | GTGATTGCAGCAGTAGGAATCGG | RT-PCR |
|  | CT229R2^r^ | CTCAATTTACACAACTCTGC | RT-PCR |
| ***CT228*** | CT228F2^f^ | GTACTACTATTAGCGGAGACG | RT-PCR |
|  | CT228R2^r^ | GCTAGCATAAGAGGATCGAG | RT-PCR |
|  | CT228F3^f^ | CTGCAATAGGTATTGCTGC | RT-PCR |
|  | CT228R3^r^ | GCTAGATAAAACTAAGCGCACATCGG | RT-PCR |
| ***CT227*** | CT227F1^f^ | GCTCCAACTCTGGAGAGC | RT-PCR |
|  | CT227R1^r^ | CACTTCTCGTCACATCTGC | RT-PCR |
| ***CT226*** | CT226F1^f^ | ATTCCAGCAAACCATTGC | RT-PCR |
|  | CT226R1^r^ | CCAATACACATTTCAACC | RT-PCR |
| ***CT225*** | CT225F1^f^ | ACAACTCCTTTATTCACC | RT-PCR |
|  | CT225R1^r^ | CATGAAATTTCGCATAAAAACCATCC | RT-PCR |
| ***CT224*** | CT224F1^f^ | AGAAGTTATATGCCAGAG | RT-PCR |
|  | CT224R1^r^ | GAATAGCCTCTTTCTCTTCC | RT-PCR |
| ***groEL*** | ED100^fƗ^ | GATGGTGTTACCGTTGCG | RT-PCR |
| ***groEL*** | ED103^rƗ^ | CCACGAATTCTGTTCACG | RT-PCR |
| ***incA*** | IncAF^f^  IncAR^r^ | ACCTTCCTACTCAGCCAATC  AATCGGCGAACTTCTTCTGC | *incA* amplification |
| ***CT228*** | CT228-2F^f^  CT228-2R^r^ | GTACCCGGGATGAGTACTACTATTAGC  GATCCCCGGGCTAAGAAGCTTGGTTAG | Verify intron insertion site |
| **intron** | GIIF^f^  GIIR^r^ | AGCGATGCCGAGAATCTG  TCTCGGAGTATACGGCTCTG | Detection of intron |
| ***CT228*** | CT228A  CT228B  CT228C  EBS universal | AAAA**AAGCTT**ATAATTATCCTTAAAACACTTTTTGGTGCGCCCAGATAGGGTG (Hind III site)  CAGAT**TGTACA**AATGTGGTGATAACAGATAAGTCTTTTTGCTTAACTTACCTTTCTTTGT (BsrGI site)  TGAACGCAAGTTTCTAATTTCGGTTTGTTTCCGATAGAGGAAAGTGTCT  CGAAATTAGAAACTTGCGTTCAGTAAAC | Retargeting of intron |
|  |  |  |  |
| ***CT228GIIaada*** | JetF^f^  JetR^r^ | CGACTCACTATAGGGAGAGCGGC  AAGAACATCGATTTTCCATGGCAG | sequencing of pJET::*CT228GIIaada* |
|  |  |  |  |
|  |  |  |  |

^f, r,^ designate Forward and Reverse primers respectively.

Ɨ - Oligonucleotide sequences from Shaw et. al Mol. Micro 2000
