# Supplementary material for: Genetic Inactivation of Chlamydia trachomatis Inclusion Membrane Protein CT228 Alters MYPT1 Recruitment, Extrusion Production, and Longevity of Infection
Source: Front Cell Infect Microbiol. 2018 Nov 30;8:415. doi: 10.3389/fcimb.2018.00415 (PMC6284022; doi:10.3389/fcimb.2018.00415)
Supplement: Supplementary file 2 [file Table_2.DOCX]

**Table S2. Pathological Raw Scores of Murine Reproductive Tracts (n = 5).**

| Infectious agent | Overall impression | Mucinous change | Hydrosalpinx | Uterine tubal dilation | Uterine luminal PMN | Representative images |
| --- | --- | --- | --- | --- | --- | --- |
| *L2-Wild type* | 1, 0, 1, 0, 3 | 2, 1, 2, 1, 1 | 0 | 1, 1, 0, 2, 3 | 0 | Fig 6A,6C |
| *L2-ΔCT228* | 2, 0, 0, 0, 0 | 0, 1, 0, 1, 0* | 0 | 2, 0, 0, 1, 0 | 0 | Fig 6B, 6D |

Histological sections of reproductive tracts (n=5/group) were examined and scored by an ACVP certified veterinary pathologist via light microscopy with the following numerical designations: 0=normal, 1= minimal change, 2=mild change, 3=moderate change, 4=severe change (mean+/-SE). *p=0.02 (Mean score for L2-wild type v. L2-ΔCT228).
