# Supplementary material for: Genetic Inactivation of Chlamydia trachomatis Inclusion Membrane Protein CT228 Alters MYPT1 Recruitment, Extrusion Production, and Longevity of Infection
Source: Front Cell Infect Microbiol. 2018 Nov 30;8:415. doi: 10.3389/fcimb.2018.00415 (PMC6284022; doi:10.3389/fcimb.2018.00415)
Supplement: Supplementary file 3 [file Data_Sheet_1.docx]

**Methods**

**Southern blot analysis of the GII*aadA* insertion in L2-ΔCT228**. Genomic DNA from the wild-type and mutant *C. trachomatis* strains was isolated using the GeneJET whole blood genomic DNA purification kit (Thermo Scientific). 1.5 µg samples were then digested using ClaI and EcoRI (Fermentas) at 37°C for two hours and then run on a 0.7% agarose gel. The gel was stained with ethidium bromide and the DNA was visualized using UV transillumination on a Bio-Rad ChemiDoc. The DNA was then transferred to a positively charged nylon membrane via capillary transfer. For DNA detection, a DIG-labeled probe was generated using the PCR DIG Probe Synthesis Kit (Roche) as directed by the manufacturer using the primers GIIFnew (5’- CGCCCAGATAGGGTGTTAAG-3’) and GIIRnew (5’- GATTCTCGGCATCGCTTTCGTTTCG-3’) and 100 pg of pDFTT295 as template to create a 358 bp DIG probe (DIG-GII). The blot was then probed with DIG-GII overnight at 42°C using DIG Easy Hyb Granules (Roche) followed by high stringency washes and probe detection with an alkaline-phosphatase conjugated anti-DIG antibody (Roche). Probe location was then visualized using the colorimetric substrate tertazolium/1H-Indol-3-ol, 5-bromo-4-chloro-dihydrogen phosphate (NBT/BCIP). Color development was allowed to proceed for 16 h, stopped by the addition of distilled water, and the blot was imaged on a Bio-Rad ChemiDoc.

**Immunofluorescence.** *C. trachomatis* L2-wild type and L2-ΔCT228 were used to infect HeLa cell monolayers on glass coverslips in 24 well plates (CellTreat Scientific, Pepperell MA) at a MOI of ~0.5 (performed in technical triplicates). At 24 and 48 hours post-infection supernatants were removed and cells were fixed in cold methanol. Recruitment of host proteins was tested with primary antibody staining to MYPT1 (United States Biological Life Sciences, Salem, MA), phospho (pSer-19)-MLC2 (Abcam, Cambridge, MA), phospho (pTyr419), Myosin IIa (ThermoFisher Scientific), Myosin IIb (ThermoFisher Scientific) and Myosin Light Chain Kinase (pTyr 471) (Santa Cruz). *Chlamydiae* were detected with anti-*Chlamydia* LPS (ThermoFisher Scientific). Fluorescent secondary antibodies, anti-mouse or anti-rabbit DyLight 594 and DyLight 488 (Jackson ImmunoResearch Laboratories, West Grove, PA) were used for indirect immunofluorescence. The entire experiment was repeated on three separate occasions (n = 3 biological replicates). Twelve images were captured per condition using a Leica DMI600B fluorescent microscope and a representative image was selected for presentation.


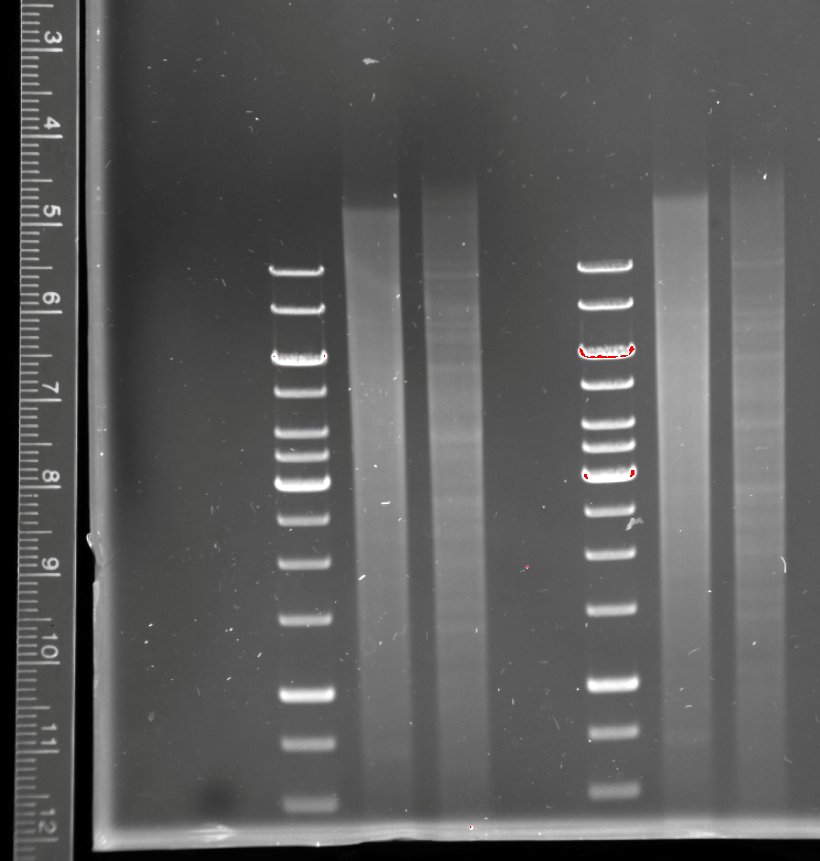

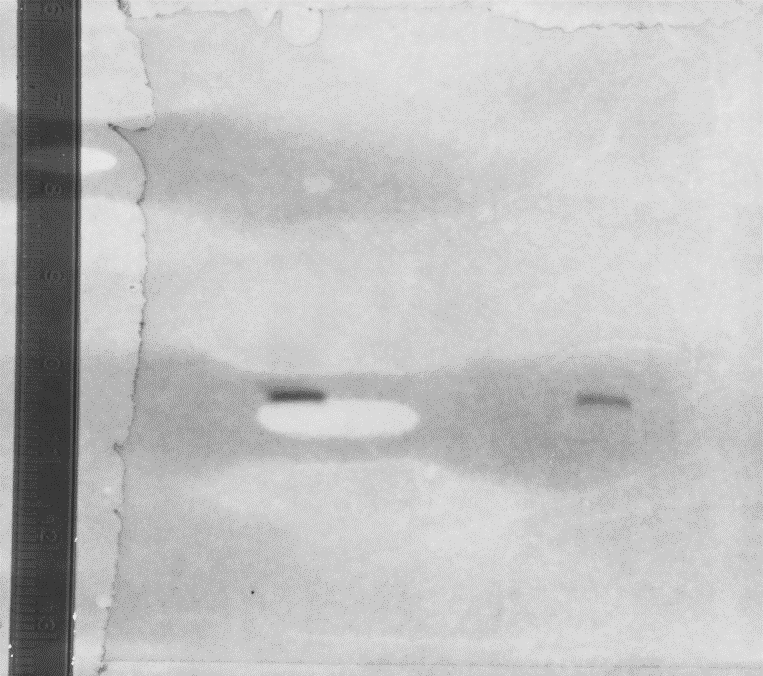


10-

8-

6-

5-

4-

3-

2-

1-

kbp

L2-wild type

L2-ΔCT228

L2-wild type

L2-ΔCT228

8-

6-

L2-ΔCT228

L2-wild type

L2-ΔCT228

L2-wild type

**A)**

**B)**

**Figure S1. Confirmation of a single GII*aadA* insertion in the L2-ΔCT228 mutant.** Genomic DNA from the wild-type parental strain and the L2-ΔCT228

mutant was digested with ClaI and EcoRI, resolved on a 0.7% agarose gel, and visualized with UV transillumination following staining with ethidium bromide (A). DNA was then transferred to a positively-charged nylon membrane and probed with a DIG-labeled GII-specific DNA probe. The probe was then detected using an alkaline-phosphatase conjugated anti-DIG antibody and the colorimetric substrate NBT/BCIP (B). Molecular weight markers are shown to the left of the gel in (A). Contrast was adjusted uniformly in panel B to aid in visualization of the probe. Results are shown for independent digestions. The expected size for the digested CT228::GII*aadA* fragment was 6.2 kbp.


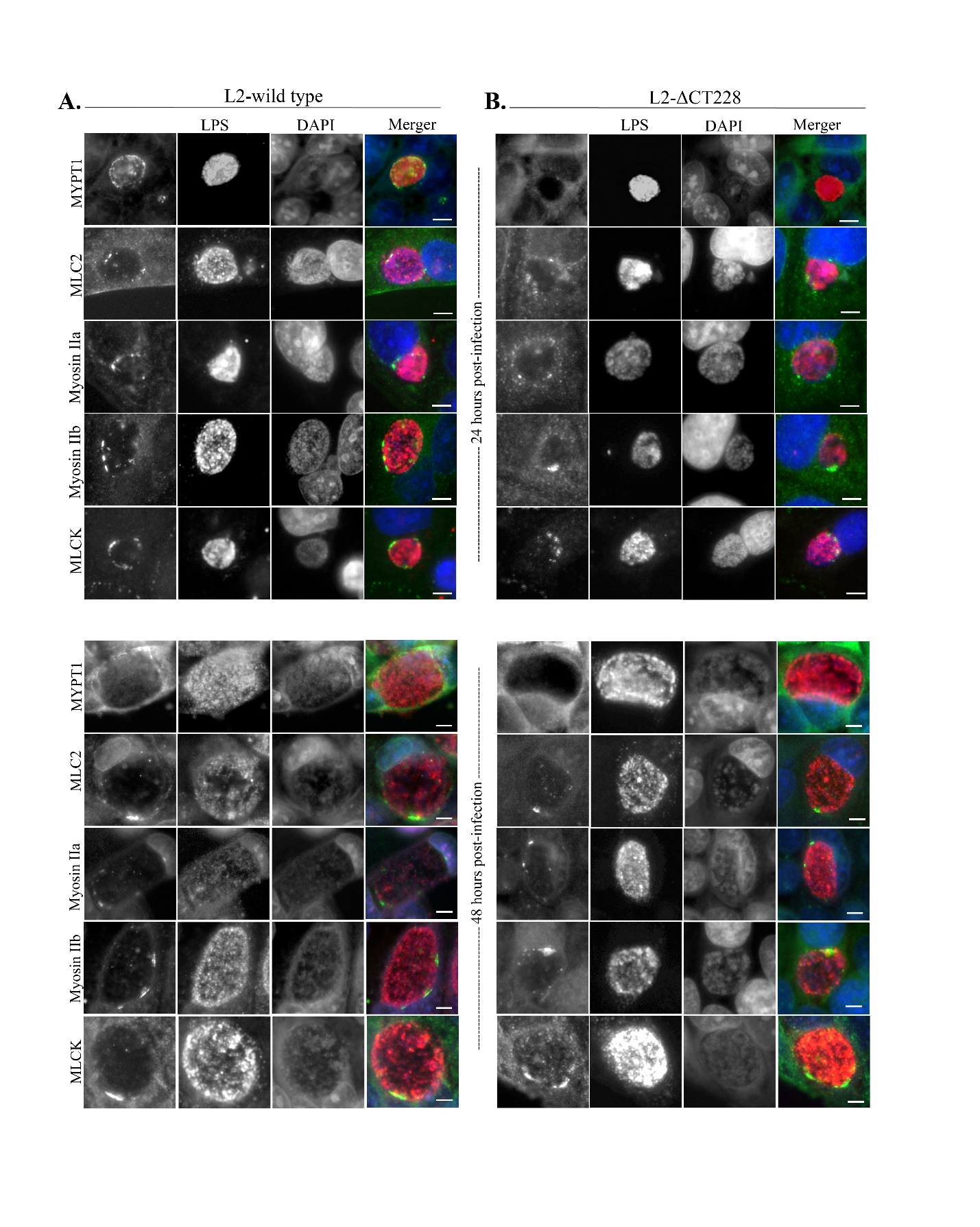


**Figure S2. Recruitment of MYPT1 and myosin phosphatase pathway proteins.** HeLa cells were infected with L2-wild type and L2-ΔCT228 for 24 and 48 hours. Cells were fixed in methanol and stained with primary antibodies to MYPT1, *Chlamydia* LPS, MLC2 (pS19), MLCK (pY471), non-muscle Myosin IIa and IIb followed by fluorescent secondary antibodies. Experiments were repeated on three separate occasions and representative images were selected. Scale bar, 10 μm.
